# Cleaning Interactions Between Crows and Sika Deer: Implications for Tick‐Borne Disease Management

**DOI:** 10.1002/ece3.70845

**Published:** 2025-02-05

**Authors:** Kanzi M. Tomita, Hiroyuki Matsuyama

**Affiliations:** ^1^ Faculty of Agriculture and Marine Science Kochi University Nankoku Japan; ^2^ Department of Infectious Diseases Hokkaido Institute of Public Health Sapporo Hokkaido Japan

**Keywords:** cleaning behavior, Ixodid ticks, parasite, web resources, zoonosis

## Abstract

Cleaning interactions between mammals and birds have been widely observed worldwide. Here, we report cleaning interactions between sika deer and crows in Japan, based on a field observations using camera traps and online research. Online research was performed on social media platforms such as X (formerly Twitter), YouTube, and personal blogs. We finally collected 27 cases of cleaning associations between sika deer and crows. Crows associated with male more than female deer and mainly pecked their heads or necks, suggesting that crows remove Ixodid ticks from the deer's surface. Given that ticks on sika deer are vectors of several zoonotic pathogens such as *Rickettsia* and *Borrelia* spp., further studies should be conducted to examine the roles of crows as biocontrol agents of ticks and tick‐borne diseases.

## Graphical abstract

We found cleaning interactions between crows and sika deer to remove ectoparasites on deer in Japan. These interactions may contribute to reducing the risk of zoonotic disease.

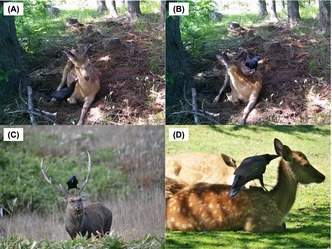

## 1 Introduction

Cleaning interactions, in which one species “cleaner” removes and consumes materials such as ectoparasites from another species “client,” are widespread in both aquatic and terrestrial ecosystems (Sazima et al. 2012; Vaughan et al. 2017). These mutualistic relationships benefit cleaner species by providing food and energy from ectoparasites, while reducing the ectoparasite load of the client species. Cleaning interactions between birds and mammals have been widely documented worldwide (Massei and Genov 1995; Sazima et al. 2012).

Cleaning interactions are expected as a driver of zoonotic disease prevention and management by reducing ectoparasite loads, which are vectors of zoonotic pathogens (Samish 2000). For example, oxpeckers (*Buphagus* spp.) are a major biocontrol agent of ticks in Africa. The daily intake of ticks by an oxpecker is roughly estimated at hundreds of adult ticks or thousands of nymphs (Samish 2000). Corvids, including crows, jays, and magpies, are sometimes observed engaging in cleaning interactions with large ungulates (Isenhart and Desante 1985; Massei and Genov 1995; Stone et al. 2024).

Here we report evidence of cleaning interactions between sika deer ( *Cervus nippon* ) and crows ( *Corvus macrorhynchos* ) in Japan, based on observations from camera traps and subsequent online research on social media platforms. Sika deer is the host of hard tick (Acari: Ixodidae) which is the vectors of several zoonotic pathogens such as *Rickettsia* and *Borrelia* spp. (Jongejan and Uilenberg 2004; Okado et al. 2021; Matsuyama et al. 2023).

## 2 Field Observation

**FIGURE 1:**
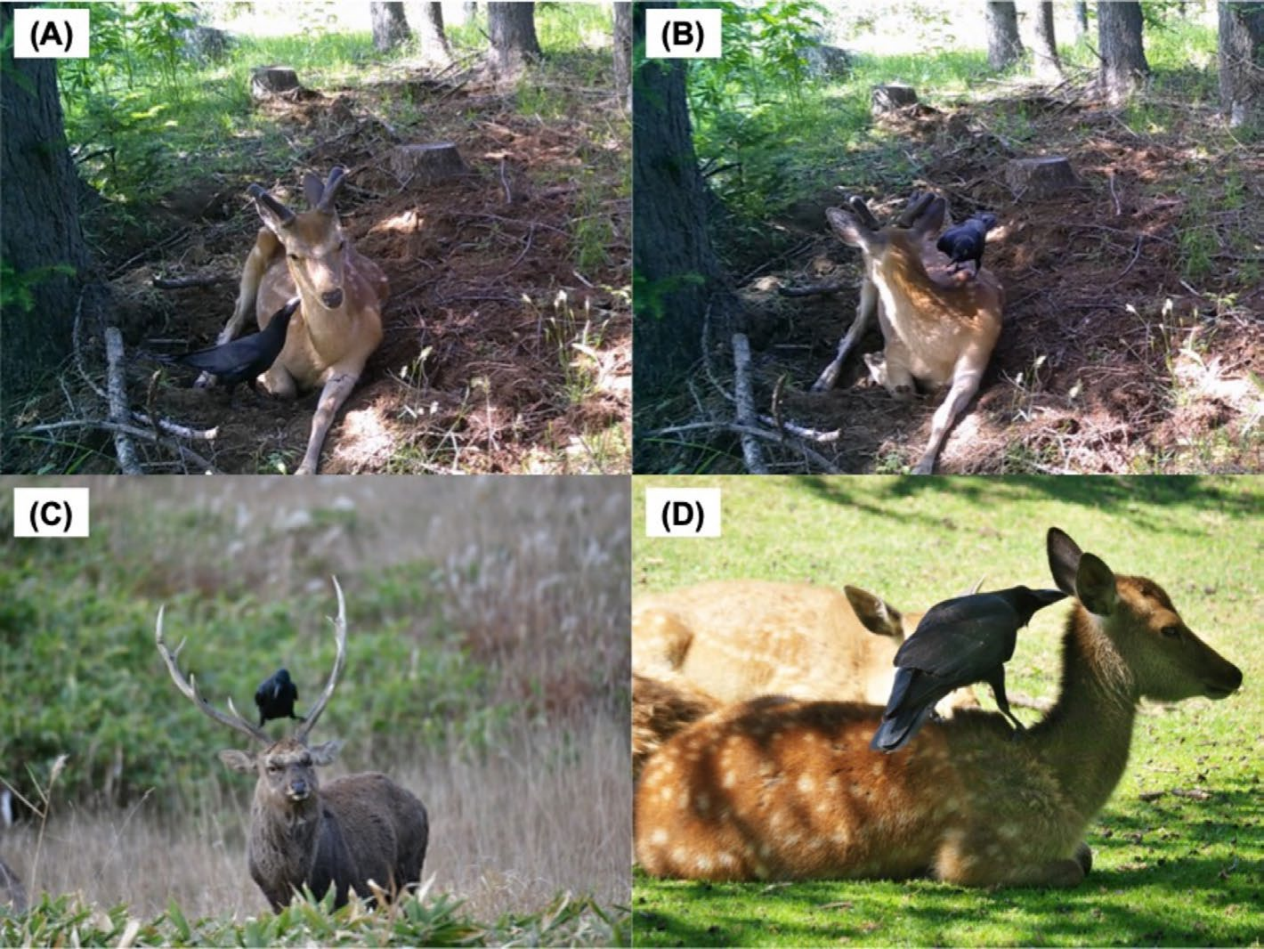
Photos of cleaning interactions between crows ( *Corvus macrorhynchos* ) and sika deer ( *Cervus nippon* ). (A, B) A large‐billed crow rides on a deer and pecks its ear on June 18, 2024 in the Shiretoko World Heritage site (44°09′ N, 145°02′ E). Detailed behavior of the crow can be watched in Videos 1 and 2; (C) A large‐billed crow rides on a male deer on November 8, 2021 in Wakkanai, northern Japan (45°24′ N, 141°40′ E) (photo credit: Ken Takeshige). (D) A large‐billed crow pecks the ear of a deer fawn on June 4, 2015 in the Nara Park (34°68′ N, 135°84′ E) (photo credit: the owner of NARA‐Photo Blog).

The cleaning interactions between a large‐billed crow and a male sika deer was found in the western parts of the Shiretoko World Heritage site, northern Japan (44°09′ N, 145°02′ E). On June 18, 2024, camera traps set by one of the authors (KMT) captured footage of this interaction (Figure 1A,B; Videos 1 and 2). The camera traps were originally intended to monitor the digging behavior of the brown bear ( *Ursus arctos* ) as it foraged for belowground cicada nymphs (Tomita and Hiura 2020; Tomita 2021). Camera traps, placed in larch ( *Larix kaempferi* ) plantations, were programmed to record 30‐s videos at 3‐min intervals.

**VIDEO 1:** A crow on deer back for a couple of seconds. Video content can be viewed at https://onlinelibrary.wiley.com/doi/10.1002/ece3.70845

**VIDEO 2:** A crow pecked to deer ear and neck with moving around deer for 20 s. Video content can be viewed at https://onlinelibrary.wiley.com/doi/10.1002/ece3.70845

The deer was first captured resting at 13:31, ruminating. At 14:12, the first video documented a crow briefly perched on the deer's back for a couple of seconds (Video 1). A subsequent video, taken at 14:15, captured a crow—likely the same individual—pecking at the deer's ear and neck for about 20 s while moving around the deer's body (Video 2). The deer remained passive throughout the interactions and left the area at 15:18. Notably, no antagonistic behavior, even from the deer were observed during the interaction. Despite 13 camera traps being placed in the study site between mid‐May and late August 2024, only one cleaning interaction was recorded.

## 3 Online Research

To collect additional evidence of cleaning interactions between crows and deer in Japan, we used online resources, including social media platforms X (formerly Twitter), YouTube, and personal blogs. Online platforms have become valuable tools for expanding scientific knowledge, particularly in natural history (Jarić et al. 2020). Wildlife photographs and videos shared on these platforms offer insights into animal behavior and species interactions (Jarić et al. 2020; Pollock et al. 2021; Noriyuki et al. 2023).

We searched for the keywords “karasu” (crow) and “sika” (deer) in Japanese on Google Images, X, and YouTube. We did not perform online research in English because such incidence are likely tweeted or posted in Japanese. Actually, when searching in English, we did not find the cases of deer and crow interactions on X. While many cases depicted crows stealing deer hair for nest‐building (*kleptotrichy* defined by Pollock et al. 2021), we focused on evidence of cleaning behavior in the following manner. For videos, we defined cleaning behavior as pecking at the deer's body or ear. We evaluated deer responses to cleaning behavior as no‐response or antagonistic. In images, we considered cleaning behavior as instances where the crow's beak made contact with the deer's fur or ear. We excluded cases of kleptotrichy and ambiguous behaviors. We also referred description about these images on each platform. We included the photos that the contributors described the incidence as cleaning behavior by crows into the database. Localities and date of photographs and movies followed uploaders' description. If such information were not described, we asked uploaders about them. Deer sex and life stage were recorded based on the appearance of deer in pictures and movies. Fisher's exact test was performed to verify sex bias of deer interacting with crows.

## 4 Results and Discussion

We found 26 cases (17 movies and 9 photos) from 20 posts of cleaning interactions between deer and crows from X (10 cases in 9 posts), YouTube (11 cases in 7 posts), and blogs via Google Images (5 cases in 4 posts) (Figure 1C,D; Table S1). The web resources were collected from Hokkaido (northernmost prefecture), Iwate, Shiga, and Nara (south‐western Japan) prefectures, suggesting that cleaning interactions between deer and crows are widespread in Japan. The source of crows' cleaning behavior was recorded from Nara Park, south‐western Japan (61.5%: 16/26). The second largest source of the behavior was recorded from Shiretoko World Heritage site (11.5%: 3/26). These sites are among the most famous to observe sika deer in Japan due to high density and legal or religious protections. Many photographers have come to both sites to take wildlife photographs (Torii and Tatsuzawa 2009). Thus, this data cannot suggest significant geographic patterns in cleaning interactions between deer and crows.

**FIGURE 2:**
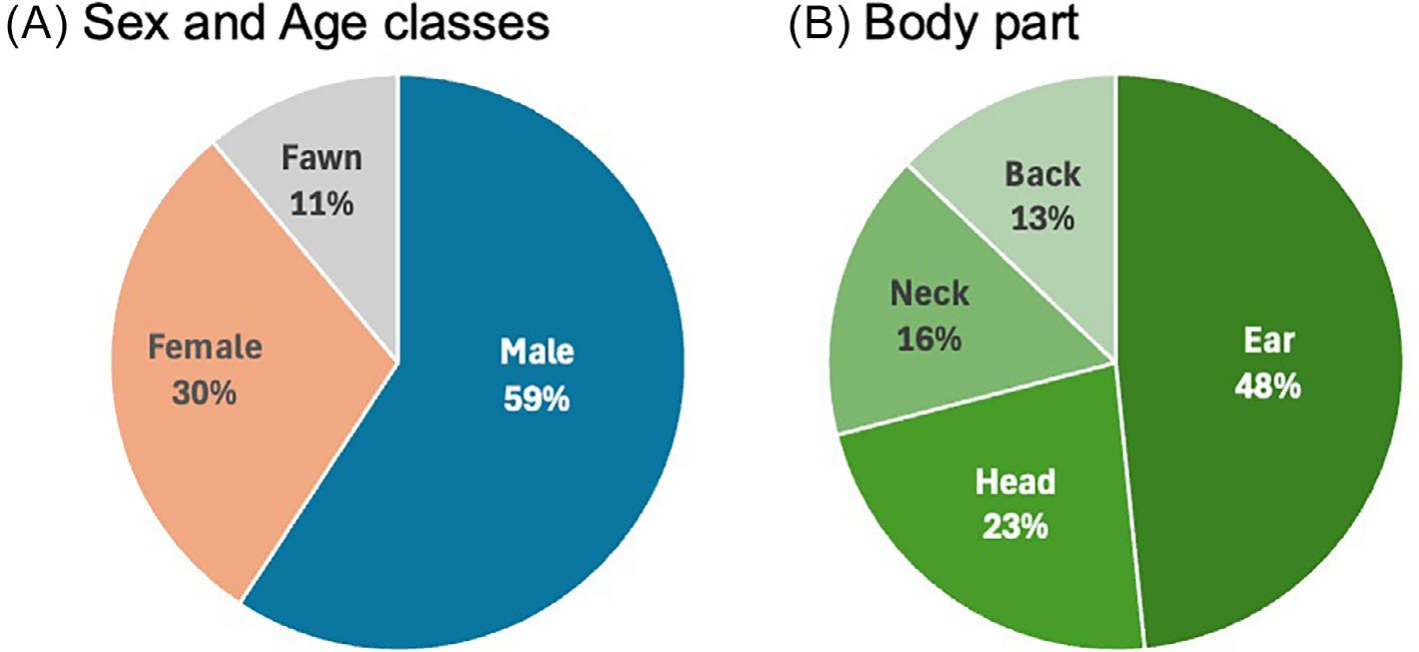
The pie charts show the proportion of (A) deer sex and age classes observed cleaning interactions with crows, and (B) body parts of deer pecked by crows.

The majority of recorded interactions occurred from spring (April) to summer (July) (81.5%: 22/27). Deer with summer fur (63.0%: 17/27) were documented more often than those with winter fur (37.0%: 10/27). For sex and age class of deer, adult male (59.3%: 16/27) was observed cleaning interactions with crows more frequently than adult female (29.6%: 8/27) and fawn (11.1%: 3/27) (Figure 2A). A Fisher's exact test revealed that male deer interacted with crows significantly more frequently than female deer (*p* = 0.042). All videos documented no antagonistic actions of deer toward cleaning behavior by crows. All recorded crows were *C. macrorhynchos* , but not *Corvus corone* . The most pecked areas were ears (48.4%: 15/31) and head (22.6%: 7/31), with fewer instances of pecking at the neck (16.1%: 5/31) and back (12.9%: 4/31) (Figure 2B).

The main ectoparasites of sika deer in Japan include louse flies (Diptera: Hippoboscidae), hard ticks, and deer lice (Psocodea: Trichodectidae) (Sato et al. 2021). Louse flies and ticks are vectors of zoonotic pathogens such as *Rickettsia* and *Bartonella* spp. (Jongejan and Uilenberg 2004; Sato et al. 2021), and louse flies were found in the highest density on the back of moose ( *Alces alces* ) (Paakkonen et al. 2010). Several studies reported higher densities of adult ticks attaching to the head or front sections (e.g., neck) of white‐tailed deer ( *Odocoileus virginianus* ), roe deer ( *Capreolus capreolus* ), and red deer ( *Cervus elaphus* ) than to their bodies and legs (Handeland et al. 2013; Poh et al. 2022). Given that the crows mainly pecked at the ear, head, and neck, they were likely to prey on ticks attaching to deer.

Sexual differences in parasite loads on sika deer would result in male‐biased cleaning behavior by crows. For ungulates such as white‐tailed deer, roe deer, and moose, males generally have higher tick density than females, even though it depends on sex and life stages in ticks (Paakkonen et al. 2010; Kiffner et al. 2011). Sexual differences in body size and immune defense are possible factors affecting male‐biased parasitism in ungulates (Vicente et al. 2007; Kiffner et al. 2011; Mysterud, Hatlegjerde, and Sørensen 2014). The immunocompetence handicap hypothesis postulates that male deer may have higher parasite loads than females due to the immunosuppressive effects of testosterone hormone (Folstad and Karter 1992; Vicente et al. 2007). Crows may particularly relax the negative effects on male deer with heavy tick burden.

This predation may also accelerate the further transmission of tick‐borne pathogens among wildlife. Various tick‐borne pathogens such as tick‐borne encephalitis virus (TBEv) or *Borrelia burgdorferi* sensu lato (s.l.) can be detected in ticks on their hosts, regardless of whether ticks have fed on a host or not (Isogai et al. 1996; Jemeršić et al. 2014; Król et al. 2020). However, it is possible that crows may also acquire pathogens through predation on pathogen‐infected ticks, given that red grouse ( *Lagopus lagopus* ) became infected the tick‐borne encephalitis virus complex by consuming virus‐infected ticks (Gilbert et al. 2004). Indeed, crows have been suspected of being infected with TBEv (Mikryukova et al. 2014) or *B. burgdorferi* s.l. (Zinck and Lloyd 2022), and being infested with various ticks (Keve, Sándor, and Hornok 2022). These pathogens have also been detected in ticks found on deer (Isogai et al. 1996; Król et al. 2020). Given that crows are urban adapter species and highly interact with human (Benmazouz et al. 2021), they may be efficient vectors of tick‐borne diseases to human. For tick‐borne disease management, further research need to understand direct pathogen transmission from deer to crows via cleaning interactions.

## Author Contributions

**Kanzi M. Tomita:** conceptualization (equal), formal analysis (lead), funding acquisition (equal), investigation (lead), methodology (lead), project administration (lead), visualization (lead), writing – original draft (lead). **Hiroyuki Matsuyama:** conceptualization (equal), formal analysis (supporting), funding acquisition (equal), investigation (supporting), methodology (supporting), validation (lead), writing – original draft (supporting), writing – review and editing (lead).

## Conflicts of Interest

The authors declare no conflicts of interest.

## Supporting information


Table S1

## Data Availability

The data that support the findings of this study are available in the Supporting Information of this article.
